# Regulator of cullins-1 (ROC1) negatively regulates the Gli2 regulator SUFU to activate the hedgehog pathway in bladder cancer

**DOI:** 10.1186/s12935-021-01775-5

**Published:** 2021-01-26

**Authors:** W. Wang, J. Qiu, P. Qu, H. Chen, J. Lan, H. Chen, L. Li, M. Gu

**Affiliations:** 1 Department of Urology, Jiangsu Provincial People’s Hospital, The First Affiliated Hospital of Nanjing Medical University, 300 Guangzhou Road, Nanjing, 210029 Jiangsu China; 2 Department of Urology, Yancheng First People’s Hospital, The Yancheng Clinical College of Xuzhou Medical University, Yancheng, 224000 Jiangsu China; 3 Department of Urology, Shanghai First People’s Hospital, School of Medicine, Shanghai Jiao Tong University, Shanghai, 200080 China; 4 Department of Haematology, Yancheng First People’s Hospital, The Yancheng Clinical College of Xuzhou Medical University, Yancheng, 224000 Jiangsu China; 5 Department of Pathology, Yancheng First People’s Hospital, The Yancheng Clinical College of Xuzhou Medical University, Yancheng, 224000 Jiangsu China; 6 Translational Medicine Center, Yancheng First People’s Hospital, The Yancheng Clinical College of Xuzhou Medical University, Yancheng, 224000 Jiangsu China

**Keywords:** Bladder cancer, ROC1, Sonic hedgehog signaling, SUFU, Gli2

## Abstract

**Background:**

The regulator of cullins-1 (ROC1) is an essential subunit in the cullin-RING ligase (CRL) protein complex and has been shown to be critical in bladder cancer cell survival and progression. This study aimed to explore the molecular mechanism of ROC1 action in the malignant progression of bladder cancer.

**Methods:**

This study utilized ex vivo, in vitro, and in vivo nude mouse experiments to assess the underlying mechanisms of ROC1 in bladder cancer cells. The expression of the components of the sonic hedgehog (SHH) pathway was determined by western blot analysis. ROC1 expression in human tumors was evaluated by immunohistochemistry.

**Results:**

ROC1 overexpression promoted the growth of bladder cancer cells, whereas knockdown of ROC1 expression had the opposite effect in bladder cancer cells. Mechanistically, ROC1 was able to target suppressor of fused homolog (SUFU) for ubiquitin-dependent degradation, allowing Gli2 release from the SUFU complex to activate the SHH pathway. Furthermore, knockdown of SUFU expression partially rescued the ROC1 knockdown-suppressed SHH activity as well as cancer cell growth inhibition. In ex vivo experiments, tissue microarray analysis of human bladder cancer specimens revealed a positive association of ROC1 expression with the SHH pathway activity.

**Conclusion:**

This study demonstrated that dysregulation of the ROC1–SUFU–GLI2 axis plays an important role in bladder cancer progression and that targeting ROC1 expression is warranted in further investigations as a novel strategy for the future control of bladder cancer.

## Background

Bladder cancer is a common malignancy; it is the fourth most commonly diagnosed cancer globally and the eighth leading cause of cancer-related deaths in males worldwide [1, 2]. Approximately 75–80% of bladder cancer patients are initially diagnosed as having nonmuscle-invasive bladder cancer (NMIBC), and a half of such patients experience recurrence within five years; in fact, up to 30% of NMIBC patients will progress to muscle-invasive bladder cancer (MIBC), and the latter initially occurs in approximately 25% of bladder cancer patients and has a poor prognosis [3]. Treatment of MIBC patients usually involves surgical resection of the tumor followed by cisplatin-based chemotherapy [3, 4]. The cisplatin-based chemotherapy causes severe toxicity and has a relatively low anticancer efficiency, with only 40–65% of metastatic bladder cancer patients showing a clinical response [3, 4]. Thus, a better understanding of the molecular mechanisms underlying bladder cancer progression and recurrence will lead to the identification of novel anticancer targets for bladder cancer therapy.

Cullin-RING ligases (CRLs) are enzymes that target proteins for ubiquitin-mediated degradation, and altered CRL activity contributes to the development and progression of human cancers, including bladder cancer [5]. CRL, also known as Skp1, cullin, or the F-box protein, belongs to the largest family of E3 ubiquitin ligases that mediate the proteasome-targeted degradation of 20% of ubiquitinated protein substrates, like the cell cycle-related, DNA replication, and signal transduction proteins as well as transcription factors [5]. Experimentally, small molecule inhibitors, such as MLN4924, have been shown to suppress CRL activation by inhibition of cullin activity, and, in turn, to effectively reduce the growth of various human cancer cells in vitro [6].

The regulator of cullins-1 (ROC1), also named as RING box protein-1 (RBX1), is a key CRL subunit that heterodimerizes with other cullins to form the CRL catalytic core [5]. ROC1 contains a small zinc-binding domain (the RING finger), which is evolutionarily conserved and is essential for embryonic development, while aberrant ROC1 expression leads to CRL dysfunction and embryonic lethality [7, 8]. ROC1 is also essential for maintenance of the genome integrity and cancer development [8–12]. Our previous studies have demonstrated that ROC1 protein is overexpressed in bladder cancer tissues and that knockdown of ROC1 expression reduces the CRL activity, thus triggering the accumulation of its specific substrates (such as p21, p27, and DEPTOR) and leading to tumor growth suppression [13, 14]. However, the mechanisms of ROC1 in the malignant proliferation of bladder cancer have not been fully elucidated. As accumulating evidence suggests an essential role of hedgehog signaling in tumor cell proliferation [15], in this study, we explored the underlying molecular mechanisms by which ROC1 regulates the sonic hedgehog (SHH) pathway in bladder cancer using in vitro and in vivo experiments. The results of this study will form the molecular basis for the future development of a novel ROC1-based targeted therapy against bladder cancer.

## Materials and methods

### Cell culture and reagents

Human bladder cancer 5637 and T24 cell lines were obtained from the Chinese Academy of Science (Shanghai, China) and maintained in Roswell Park Memorial Institute medium-1640 (Gibco, Gaithersburg, MD, USA) supplemented with 10% fetal bovine serum (Gibco) and 1% penicillin–streptomycin in a humidified 5% CO_2_ environment at 37 °C. Cycloheximide, the SHH signaling activator smoothened (SMO) agonist (SAG), the SMO antagonist GDC0449, and the Nedd8-activating enzyme inhibitor MLN4924 were purchased from Sigma-Aldrich (St. Louis, MO, USA), dissolved in dimethyl sulfoxide (Sigma-Aldrich), and stored at − 20 °C as stock solutions. G418 was also from Sigma-Aldrich. The recombinant plasmid carrying hemagglutinin (HA)-tagged ubiquitin cDNA was purchased from Invitrogen (Shanghai, China).

### Establishment of stable ROC1-overexpressed or -silenced bladder cancer cell lines

To establish a stable ROC1-overexpressed bladder cancer cell subline, we subcloned the full-length wild-type human ROC1 cDNA into the pcDNA3.1 vector (Invitrogen, Shanghai, China), named as pcDNA3.1-ROC1. After DNA sequence confirmation, this recombinant plasmid or pcDNA3.1 vector-only plasmid was transfected into bladder cancer cells using Lipofectamine 2000 (Invitrogen) for 48 h, and the cells were then cultured in G418-selecting cell culture medium at 100 μg/mL for 14 days. After that, individual G418-resistant monoclonal cells were selected and expanded in the 100 μg/mL G418-selective medium. The stable cell sublines were named as p-ROC1 or p-CONT. Furthermore, to knock down ROC1 or suppressor of fused homolog (SUFU) expression, we purchased siRNA oligonucleotides targeting ROC1 or SUFU from Invitrogen (Shanghai, China) and transfected them into bladder cancer cells, according to the manufacturer’s instructions. The ROC1 siRNA sequence was 5ʹ-GACTTTCCCTGCTGTTACCTAA-3ʹ; the SUFU siRNA sequence was 5ʹ-GCCATGACAATCGGAAATCTA-3ʹ; and the scrambled control siRNA sequence was 5ʹ-ACGTGACACGTTCGGAGAA-3ʹ.

### Cell viability and colony formation assays

Changed cell viability was assessed by using the Cell Counting Kit-8 kit (Beyotime, China), as previously described [13]. For the colony formation assay, tumor cells were seeded in triplicate into 35-mm culture dishes at a density of 400 cells (for 5637 tumor cells) or 1000 cells (for T24 cells) per well and cultured for 9 days. The cells were fixed and stained with crystal violet in 50% methanol, and the number of cell colonies with more than 50 cells was counted.

### Flow cytometry cell cycle distribution assay

Both ROC1-overexpressed and siRNA-transfected bladder cancer cells were detached from the cell culture dishes and fixed in ice-cold 70% ethanol overnight. One day later, the cells were washed twice with ice-cold phosphate-buffered saline (PBS), then stained with propidium iodide (Sigma-Aldrich) solution (20 mg/mL) for 5 min, and finally analyzed by using a BD FACScan flow cytometer (BD Biosciences, San Diego, CA, USA).

### Quantitative reverse transcriptase-polymerase chain reaction (qRT-PCR)

Total RNA was isolated with the Trizol reagent (Invitrogen) and reversely transcribed into cDNA with a PrimeScript Reverse Transcription kit (Takara, China), according to the manufacturers’ protocols. The resultant cDNA samples were then amplified using a 7300 Real-Time PCR system (Applied Biosystems, Foster City, CA, USA) with the SYBR Green master mix kit (Takara, Dalian, China) for detection of different genes using gene-specific primers (the detailed DNA sequences of each primer used in this study are available upon request). All measurements were performed in triplicate and quantified using the 2^−∆∆^Ct method.

### Western blot and co-immunoprecipitation-Western blot

After the cells were subjected gene transfection or drug treatments, cell lysates were prepared and quantified according to a previous study [14]. The western blot was carried out as described previously [13], while the co-immunoprecipitation kit (Cat. #26419) from Thermo Scientific (Waltham, MA, USA) was used according to the manufacturer’s instructions with the following antibodies: anti-ROC1 (Abcam, Cambridge, MA, USA); anti-cyclin D1, anti-Cdc25c, anti-SUFU, anti-Gli1, anti-GAPDH, and anti-HA (Abcam, Hangzhou, China); and anti-Gli2 (Santa Cruz Biotechnology, Santa Cruz, CA, USA).

### Immunofluorescence staining

Immunofluorescence staining was performed to assess Gli2 expression in cells, as described previously [13]. Briefly, cells were grown on coverslips, fixed, and permeabilized, and then they were incubated with a primary antibody against Gli2 (Santa Cruz Biotechnology) followed by incubation with the Alexa 548-conjugated anti-rabbit IgG (Invitrogen, Carlsbad, CA, USA). Subsequently, the cells were counterstained by using 4,6-diamidino-2-phenylindole (Sigma) and analyzed under a Zeiss LSM500 confocal microscope (Zeiss International, Oberkochen, Germany).

### In vivo tumor cell xenograft assay

An orthotopic tumor model of bladder cancer was used. In particular, tumor cells were cultured to reach 70–80% confluency, harvested, resuspended in PBS, and then mixed with Matrigel (Invitrogen) at a 1:1 vol/vol ratio. Next, mice (6-week-old, male, athymic, BALB/C nu/nu; n = 10 per group) were anesthetized by using 40 mg/kg sodium pentobarbital, and a small lower abdominal incision was made to expose the bladder for tumor cell injection. Tumor cells were then injected into the bladder wall using a 28-gauge needle; thereafter, the injection site was pressed with a cotton swab for 30 s, and the skin incision was then closed with the absorbable line. Tumor cell xenograft formation and growth were assessed by using the whole-body fluorescence imaging system weekly, with a Spectrum in vivo imaging system (Promega, Madison, WI, USA) with 470-nm excitation from an MT-20 light source. The emitted fluorescence signal was collected by using appropriate filters on a DP70 CCD camera and processed for contrast and brightness with Paint Shop Pro 8 (Corel, Ottawa, ON, Canada). Twelve weeks after the nude mice were inoculated with the pROC1 or pCONT tumor cells, the mice were sacrificed, and the xenograft tissues were resected. This study protocol was approved by the Animal Care and Use Committee of Yancheng First People’s Hospital (Jiangsu, China) and carried out following the Guidelines for the Care and Use of Laboratory Animals issued by the Chinese Council on Animal Research.

### Human bladder tissue samples and immunohistochemistry

Bladder cancer tissue specimens were retrospectively collected from 93 bladder cancer patients who were cared for at Yancheng First People’s Hospital (Jiangsu, China) between January 2010 and May 2015. The patients included 79 males and 14 females with a median age of 67 years old (range: 45–87 years old); 43 of the patients underwent a transurethral resection, 12 underwent a partial cystectomy, and 38 underwent a radical cystectomy. Their tumor grade and stage were classified according to the World Health Organization 1973 criteria and the American Joint Committee on Cancer 2002 Tumor, Node, Metastasis system. This study of human subjects was approved by the Medical Ethics Committee of Yancheng First People’s Hospital (Permit Number: 2013KY004), and informed consent was obtained from each patient before enrolling into this study.

Paraffin-embedded tissue blocks were retrieved from the Pathology Department and used for the preparation of the tissue microarray and then immunostained with a primary antibody against ROC1 (Abcam), SUFU (Abcam), Ki67 (Boster, Wuhan, China), or Gli2 (Boster), according to our previous study [13].

### Statistical analysis

The western blot band intensities were quantified by using Image J software (National Institutes of Health, Bethesda, MD, USA). The data were expressed as means ± standard error of the mean (SEM) and statistically analyzed by using SPSS 13.0 (SPSS, Inc., Chicago, IL, USA). For multi-group comparisons, the Bonferroni t-test was used after one-way analysis of variance. Meanwhile, for two-group comparisons, the Student’s *t*-test was used. The correlation between ROC1 or SUFU and Gli2 expression was assessed by using Pearson’s χ^2^ test. A *p* value < 0.05 was considered statistically significant.

## Results

### ROC1 induces bladder cancer cell growth in vitro and in vivo

**Fig. 1:**
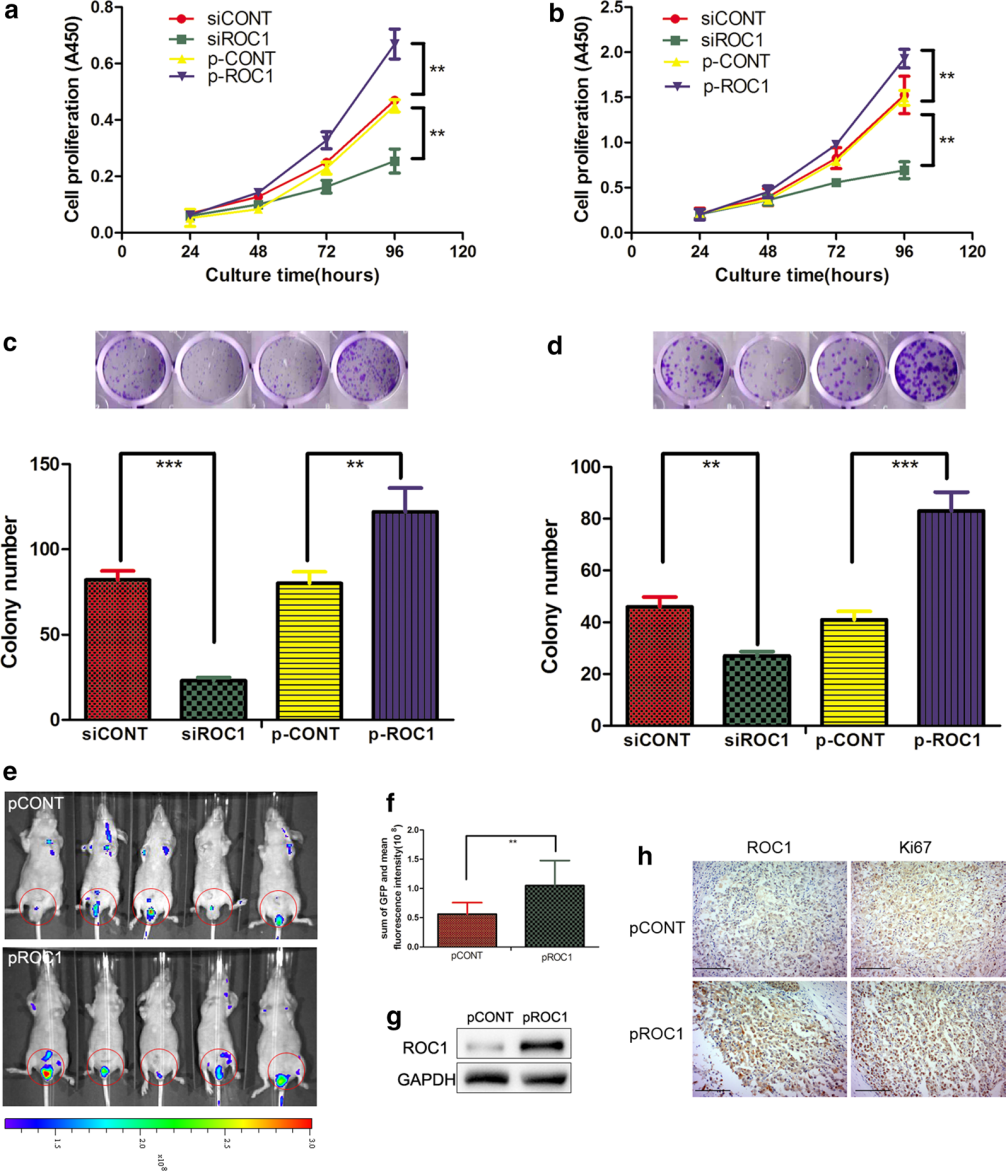
ROC1 induction of bladder cancer cell proliferation in vitro and in vivo*.* **a**, **b** Cell viability CCK8 assay. Stable ROC1-overexpressed bladder cancer 5637 (**a**) and T24 (**b**) cells and transient ROC1 siRNA-transfected 5637 (**a**) and T24 (**b**) cells were grown and subjected to the cell viability assay. **c**, **d** Colony formation assay. Stable ROC1-overexpressed bladder cancer 5637 (**c**) and T24 (**d**) cells and transient ROC1 siRNA-transfected 5637 (**c**) and T24 (**d**) cells were grown and subjected to the tumor cell colony formation assay. **e** Nude mouse orthotopic tumor cell xenograft assay. Mice were inoculated with the pROC1- or pCONT-transfected bladder cancer T24 cells and monitored with an in vivo imaging system (the blue-to-red color represents the low-to-high intensity of tumor burden) over the time period of the experiment. **f** Quantitation of the fluorescence intensity in mice after they were injected with pROC1- or pCONT-transfected cells. **g** Western blot. Tumor xenografts were taken and subjected to western blot analysis of ROC1 protein. **h** Immunohistochemistry. Tumor xenografts were taken and subjected to immunohistochemistry. Cells with a brown color were considered immunopositive. Representative results of three independent experiments are shown as means ± SEM; **P < 0.01, ***P < 0.001. Scale bar, 50 µm

To evaluate the role of ROC1 in bladder cancer proliferation, we first assessed the oncogenic activity of ROC1 in bladder cancer 5637 and T24 cells by stable transfection of ROC1 cDNA (p-ROC1) or small interfering RNA (siRNA) (siROC1), while the empty vector (p-CONT) and the negative control siRNA (siCONT) were used as controls, respectively (Additional file 1: Fig. S1). In these two cell lines, knockdown of ROC1 expression reduced tumor cell growth (Fig. 1a, b) and their colony forming potential (Fig. 1c, d). In contrast, ectopic overexpression of ROC1 significantly induced the growth and colony forming capacity of both cell lines (Fig. 1a–d) in vitro.

Subsequently, our in vivo orthotopic bladder cancer cell xenograft model in nude mice also showed that overexpression of ROC1 resulted in a significant acceleration of tumor cell xenograft growth (Fig. 1e, f), while our western blot analysis of the tumor cell xenografts confirmed ROC1 upregulation in the pROC1 group of mice compared with the vector-control group of mice (Fig. 1g). Moreover, immunohistochemical staining of the Ki67 antibody also indicated that ROC1 overexpression enhanced the percentage of proliferating xenografted cells (Fig. 1h).

### ROC1 upregulates the cell cycle progression of bladder cancer cells

**Fig. 2:**
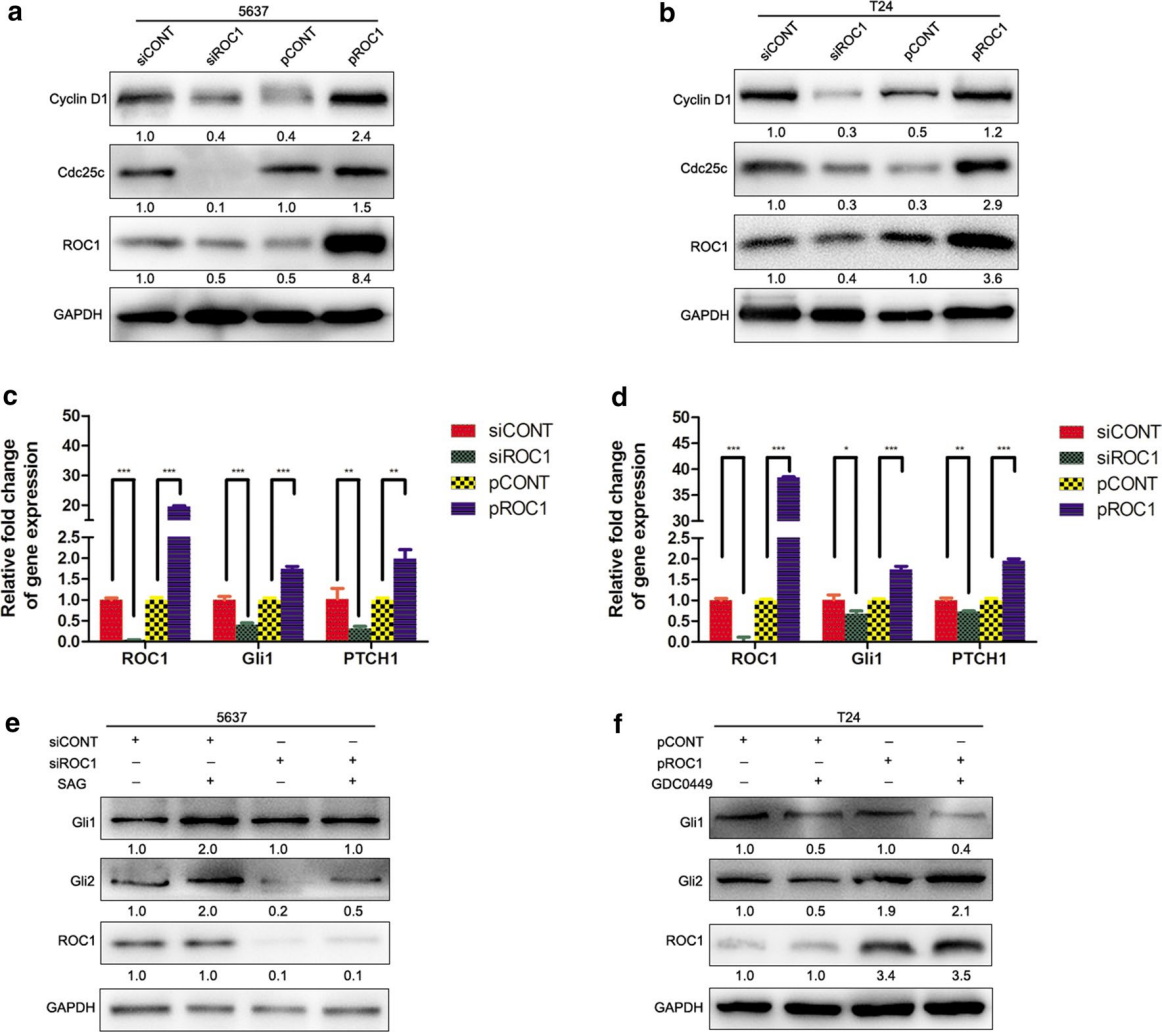
ROC1 regulation of tumor cell growth through hedgehog signaling. **a**, **b** Western blot. Stable ROC1-overexpressed and transient ROC1 siRNA-transfected 5637 (**a**) and T24 (**b**) cells were grown and subjected to western blot analysis of cyclin D1 and Cdc25c expression. **c**, **d** qRT-PCR. Stable ROC1-overexpressed and transient ROC1 siRNA-transfected 5637 and T24 cells were grown and subjected to qRT-PCR analysis of Gli1 and PTCH1. **e**, **f** Western blot. Transient ROC1 siRNA-transfected 5637 cells were treated with SAG (**e**), stable ROC1-overexpressed T24 cells were treated with the hedgehog signaling pathway inhibitor GDC0449 (**f**), and then the cells were subjected to western blot analysis of Gli1 and Gli2. Bars, SEM; *P < 0.05, **P < 0.01, ***P < 0.001

Our flow cytometric analysis of the cell cycle distribution showed that knockdown of ROC1 expression in bladder cancer 5637 and T24 cells increased the number of cells in the G2/M phase of the cell cycle (Additional file 2: Fig. S2). Moreover, the levels of cell cycle-regulated proteins were also changed, i.e., the expression of cyclin D1 and Cdc25c was markedly downregulated after knockdown of ROC1 expression in both 5637 and T24 cells, whereas ROC1 overexpression upregulated the protein levels of cyclin D1 and Cdc25c (Fig. 2a, b).

### ROC1 induces bladder cancer cell proliferation via the hedgehog pathway

Accumulating evidence suggests an essential role of hedgehog signaling in tumor cell proliferation [16], thus, we first assessed the levels of the key molecules of Gli1 and PTCH1 mRNA and found that knockdown of ROC1 expression was able to downregulate the expression of Gli1 and PTCH1, whereas ROC1 overexpression could upregulate the expression of Gli1 and PTCH1, compared with those of the corresponding controls (Fig. 2c, d). Moreover, knockdown of ROC1 expression reduced the expression of Gli2, but not Gli1, in the 5637 cell line (Fig. 2e and Additional file 3: Fig. S3). In addition, the hedgehog signaling activator SAG (a SMO agonist) was able to rescue such downregulation (Fig. 2e and Additional file 3: Fig. S3). In contrast, ROC1 overexpression upregulated Gli2 expression in T24 cells, which was attenuated by the SMO antagonist GDC0449 (Fig. 2f and Additional file 3: Fig. S3).

### ROC1 activates hedgehog signaling through SUFU deregulation

**Fig. 3:**
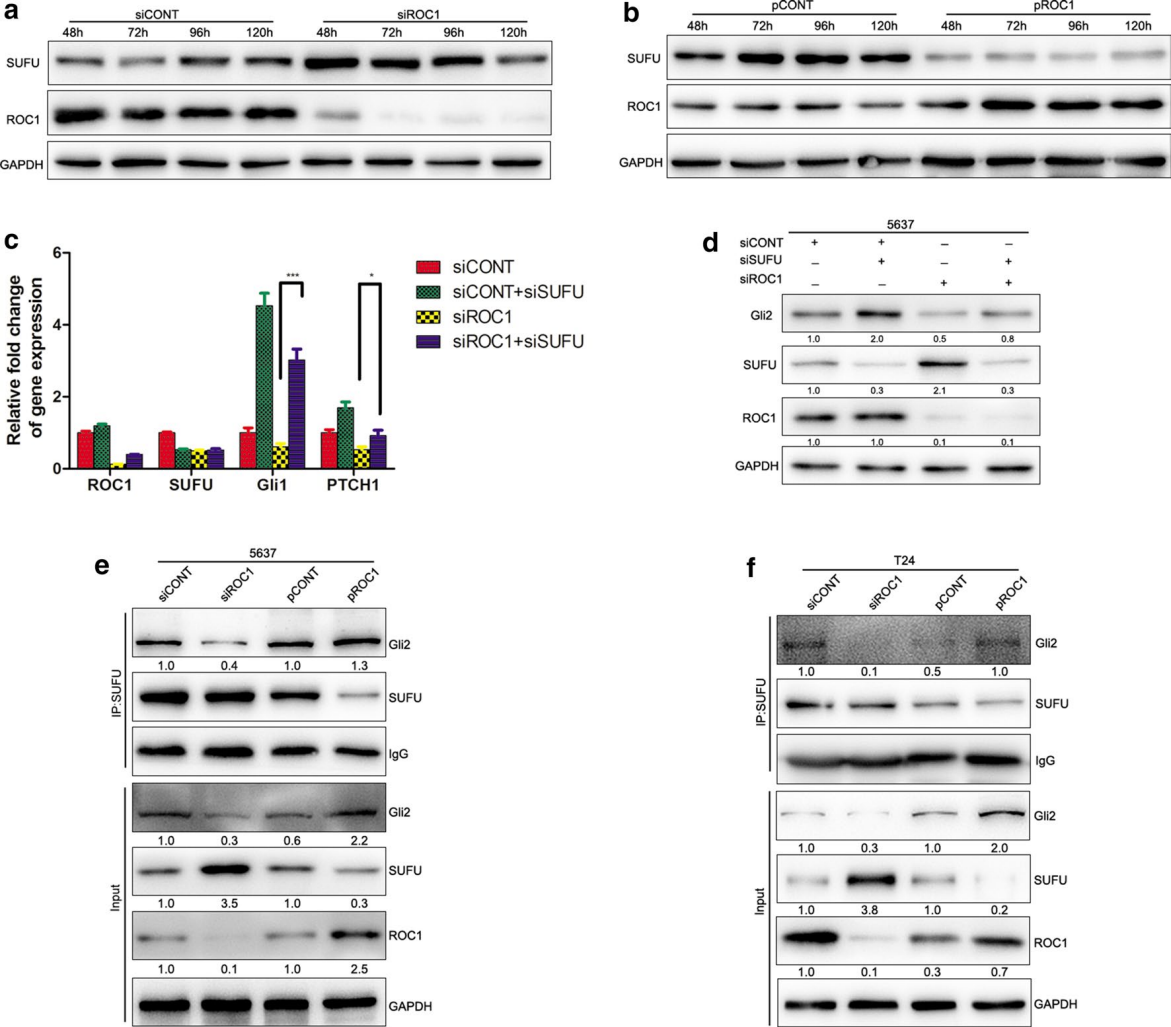
ROC1 modulation of SUFU protein levels. **a**, **b** Western blot. Expression of SUFU protein in 5637 (**a**) and T24 (**b**) cells after transfection with siROC1 or plasmid-ROC1 for 48, 72, 96, and 120 h. **c** qPCR. Bladder cancer 5637 cells were cotransfected with siRNA targeting ROC1 and SUFU and then subjected to qRT-PCR analysis of Gli1 and PTCH1 mRNA. **d** Western blot. Bladder cancer 5637 cells were cotransfected with siRNA targeting ROC1 and SUFU and then subjected to western blot analysis of Gli2 and SUFU protein. **e**, **f** Immunoprecipitation. Immunoprecipitation of SUFU from 5637 cells (**e**) and T24 cells (**f**) that were transfected with either siROC1 or pROC1. Nonspecific rabbit immunoglobulin G (IgG) was used as a negative control. Cell lysates were subjected to western blot analysis. Bars, SEM; *P < 0.05, ***P < 0.001

To explore the molecules that are primarily responsible for hedgehog activation, we assessed SUFU expression in ROC1-knocked down or ROC1-overexpressed bladder cancer cells. SUFU protein acts as a CRL substrate and a naturally occurring hedgehog inhibitor [17, 18]. Our data showed that the expression of SUFU protein was significantly elevated in the ROC1-knocked down 5637 cancer cells compared with that of the control cells (Fig. 3a). In contrast, ROC1 overexpression reduced the SUFU levels in the T24 cell line (Fig. 3b).

To verify the role of SUFU in the regulation of the hedgehog pathway upon ROC1 expression, we performed experiments using the simultaneous siRNA knockdown of both SUFU and ROC1 expression in 5637 cells. We found that knockdown of SUFU expression markedly attenuated the ROC1 knockdown-inhibited hedgehog pathway, as evident by the reduction of the Gli1 and PTCH1 mRNA levels (Fig. 3c). Our cell viability assay also demonstrated that knockdown of SUFU expression was able to partially restore tumor cell growth upon ROC1 knockdown in 5637 and T24 cells (Additional file 4: Fig. S4). These data suggest that SUFU plays a key role in transactivation of the hedgehog pathway triggered by ROC1.

### The ROC1–SUFU–Gli2 axis regulates the hedgehog pathway in bladder cancer cells

SUFU, the key subunit of the hedgehog pathway, can bind to Gli1/Gli2 to form the SUFU-Gli1/Gli2 complex and functions to prevent it from nuclear translocation, resulting in cytoplasmic retention. Thus, we first performed the co-immunoprecipitation assay to assess which Gli family member interacts with SUFU and found an interaction between SUFU and Gli1/Gli2 in 5637 and T24 cells (Additional file 5: Fig. S5).

To assess whether the ROC1-regulated Gli2 expression or activity was through the ROC1 interaction with SUFU, we performed an SUFU immunoprecipitation assay with cells transfected with either siROC1 or pROC1. Our data showed that knockdown of ROC1 expression suppressed the SUFU-mediated Gli2 expression, whereas ROC1 overexpression promoted the SUFU-mediated Gli2 expression (Fig. 3e, f). Our data indicate that ROC1-regulated hedgehog activation is through the SUFU–Gli2 axis.

### ROC1 regulates SUFU degradation in the ubiquitination-dependent manner

**Fig. 4:**
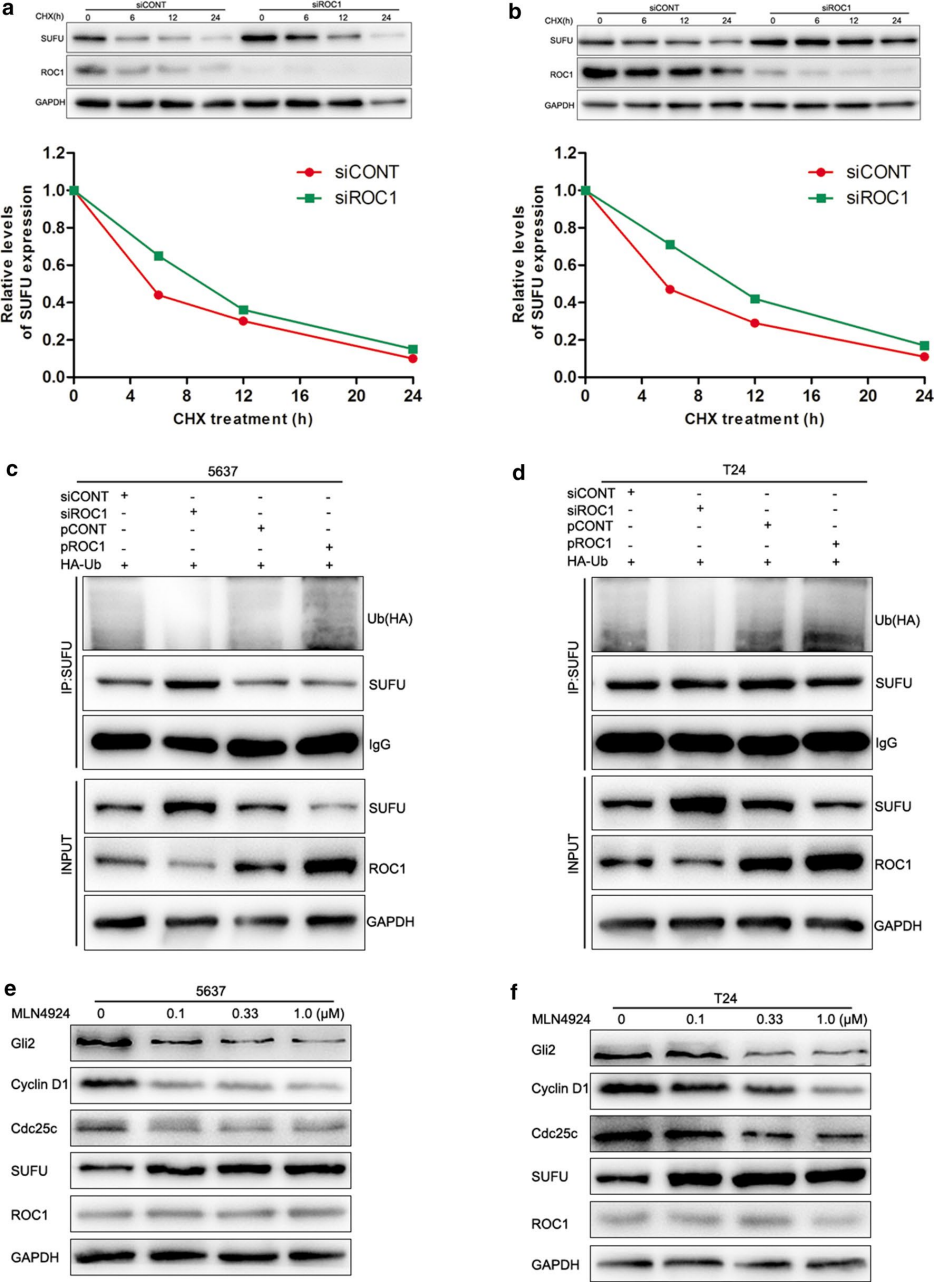
ROC1 regulation of SUFU ubiquitination for degradation. **a**, **b** Western blot. ROC1-knocked down and ROC1-overexpressed 5637 (**a**) and T24 (**b**) cells were treated with cycloheximide (CHX) for the indicated time points and then subjected to western blot analysis of SUFU protein. The graph shows the quantified data of the western blots depicted in the bottom panel. **c**, **d** Coimmunoprecipitation. Detection of ubiquitylated SUFU in 5637 (**c**) and T24 (**d**) cells cotransfected with HA-tagged ubiquitin (Ub) along with either siROC1 or pROC1 by western blot analysis of the cell lysates. Immunoprecipitation of HA antibody of nonspecific rabbit immunoglobulin G (IgG) was used as a negative control. **e**, **f** Western blot. 5637 (**e**) and T24 (**f**) cells were treated with the CRL inhibitor MLN4924 at different concentrations and then subjected to western blot analysis of Gli2, cyclin D1, Cdc25c, and SUFU proteins

Knockdown of ROC1 expression using siROC1 enhanced the SUFU protein level (Fig. 3a), while the mRNA level of SUFU remained unchanged or even slightly deceased after knockdown of ROC1 expression (Fig. 3c). These data indicate that ROC1-downregulated SUFU expression could be through a protein synthesis perturbation or a protein post-translational mechanism. Thus, we treated tumor cells with the protein translation blocker cycloheximide and then assessed the SUFU turnover rate at the same setting. As shown in Fig. 4a, b, we found that knockdown of ROC1 expression significantly delayed the SUFU turnover and prolonged the half-life of SUFU protein in both 5637 and T24 cells. We then postulated that the ubiquitination modification may regulate ROC1-related SUFU degradation, so we checked the level of ubiquitination. As shown in Fig. 4c, d, exogenous overexpression of HA-tagged ubiquitin in transfected cells did interact with SUFU, while ROC1 overexpression significantly promoted polyubiquitination of SUFU. However, knockdown of ROC1 expression using ROC1 siRNA strongly suppressed the SUFU polyubiquitination in both 5637 and T24 cells. These findings indicate that ROC1 mediates and targets SUFU for ubiquitination and degradation in bladder cancer cells.

### CRL inactivity inhibits hedgehog signaling in bladder cancer cells

MLN4924, a selective inhibitor of Nedd8-activating enzyme [6], was able to block the cullin neddylation required for the CRL activity in cells [19]. Our data showed that treatment with MLN4924 significantly reduced the viability (Additional file 6: Fig. S6A and B) and colony formation of 5637 and T24 cells in a dose-dependent manner (Additional file 6: Fig. S6C and D). Furthermore, treatment with MLN4924 for 72 h also significantly reduced the expression of cyclin D1 and Cdc25c in both cell lines, while the hedgehog pathway activity was also inhibited, as demonstrated by the decrease in Gli2 expression (Fig. 4e, f). These results suggest that inhibition of CRL activity has a similar effect as ROC1 at blocking hedgehog signaling in bladder cancer cells.

### ROC1 expression correlates with SUFU and Gli2 expression

**Fig. 5:**
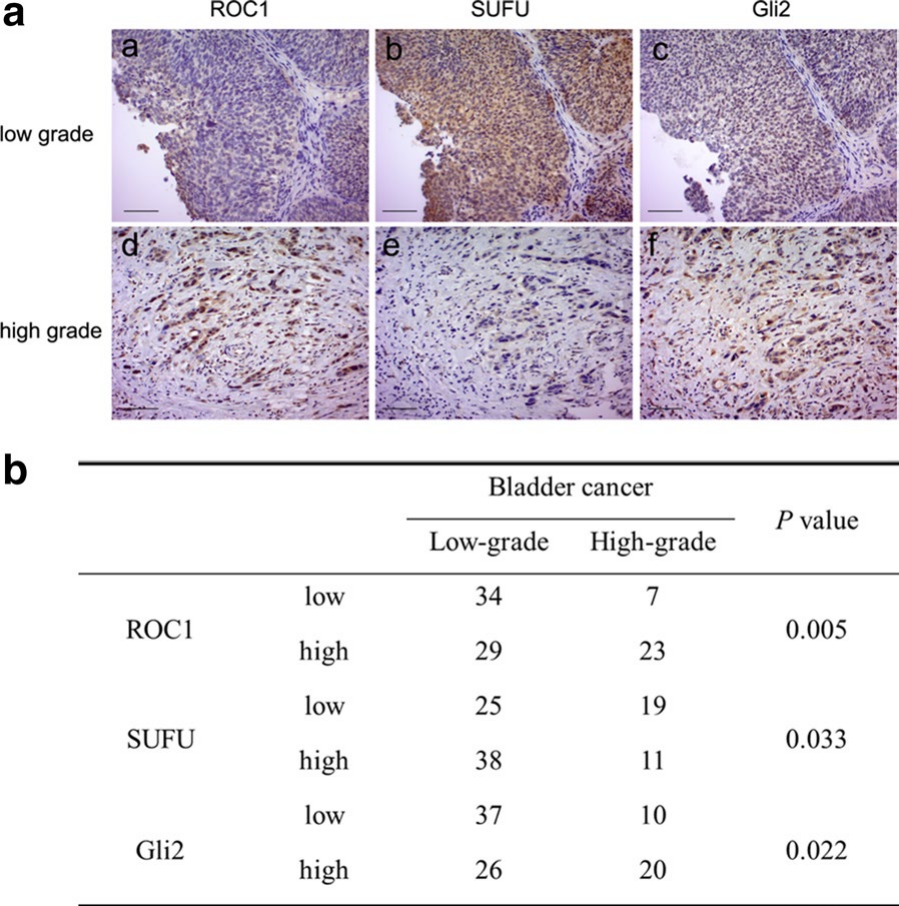
ROC1 expression in human bladder cancer tissues. **a** The expression of ROC1, SUFU, and Gli2 proteins was immunohistochemically analyzed in human bladder cancer tissues. Representative immunohistochemical images of low-grade or high-grade cancer are shown. **b** Association of ROC1, SUFU, and Gli2 expression with the clinicopathological grade. The expression levels were divided into two categories (low vs. high) according to their immunoreactivity scores and were associated with the cancer pathological grade (low-grade vs. high-grade) by using the χ^2^ test. Scale bar, 50 µm

To explore the association of ROC1, SUFU, and Gli2 expression with the tumor clinicopathological grade, we analyzed their expression immunohistochemically in human bladder cancer tissues. We then divided their expression levels into two categories (low vs. high) and found that ROC1 and Gli2 expression was low, but SUFU expression was high in the low-grade bladder cancer tissues compared with those in high-grade tumors (Fig. 5a). Moreover, the ROC1 and SUFU expression levels were associated with the tumor pathological grade (Fig. 5b), whereas the ROC1 expression was inversely associated with the SUFU expression (*P* = 0.014) but positively associated with the Gli2 expression (*P* = 0.001). Collectively, bladder cancer tissues with low ROC1 expression had high SUFU and low Gli2 expression levels.

## Discussion

Our present study demonstrated that ROC1 overexpression promoted the growth of bladder cancer 5637 and T24 cell lines in vitro and enhanced the growth of mouse orthotopic xenografts in nude mice. In contrast, knockdown of ROC1 expression had the opposite effects. Mechanistically, ROC1 targeted SUFU for ubiquitin-dependent degradation to release Gli2 from the SUFU complex, in turn activating the hedgehog pathway. These findings suggest that ROC1 plays an important role in the malignant proliferation of bladder cancer and that targeting ROC1 expression may help to control bladder cancer progression or recurrence in the future.

Dysregulation of cell proliferation is a landmark during tumorigenesis and progression, while induction of cell cycle arrest is an important strategy for the development of anticancer drugs that effectively control human cancers [20]. Previous studies by others and us have demonstrated that knockdown of ROC1 expression is able to induce cancer cell G2/M arrest, autophagy, and senescence [10, 13, 21]. In the current study, we revealed that exogenous overexpression of ROC1 promoted bladder cancer cell proliferation, whereas knockdown of ROC1 expression inhibited tumor growth through induction of G2/M arrest. Consistent with our current findings and further supporting the importance of CRL in the regulation of tumor cell proliferation, the suppression of CRL activity using FBXO22 silencing inhibited cancer progression through targeting of HDM2 for degradation in breast cancer [12]. Mechanistically, DNA damage is the most common cause of inducing cell G2/M arrest [20]. Indeed, ROC1 silencing was able to trigger the DNA damage response as a result of DNA re-replication [8]. Furthermore, a recent study has shown that ROC1 silencing leads to deficiency in DNA double-strand break repair by exonuclease 1 excessive degradation [22]. Taken together, we postulate that knockdown of ROC1 expression inhibited cancer cell proliferation by induction of cell cycle G2 arrest due to DNA damage.

The hedgehog pathway is important in cell differentiation and embryonic development [15]. A variety of cancer types also have been linked to the aberrant activity of the hedgehog signaling pathway [15, 16]. Gli1 and Gli2 are two important transcriptional factors in the hedgehog pathway, e.g., Gli1 acts as a transcriptional activator and is regulated by Gli2, while Gli2 acts as a strong activator of hedgehog signaling [15]. Upon stabilization, Gli2 translocates into the cell nucleus to promote transcription of both Gli1 and Gli2 and other target genes [23]. In general, Gli2 could play a more important role in bladder cancer [24]. In the current study, we demonstrated that knockdown of ROC1 expression inhibited Gli2 expression but not Gli1 expression, whereas ROC1 overexpression promoted Gli2 expression in bladder cancer cells. These findings suggest that ROC1 regulates the hedgehog pathway in a Gli2-dependent manner. Notably, another study has revealed that the SPOP–CUL3–RBX1 E3 ubiquitin ligase complex can activate the hedgehog pathway through ZBTB3 degradation [25]. Thus, CRL inactivation suppresses hedgehog activity via different pathways, but the exact mechanism remains unknown. We suppose that the mechanism may depend on the cell line or the cell treatment.

SUFU, a key negative regulator of the hedgehog pathway, can bind to Gli to inhibit the hedgehog pathway activity [17], while SUFU protein can be degraded by the CRL^Fbxl17^ E3 ubiquitin ligase [18]. In the current study, we demonstrated that SUFU accumulation inactivated the hedgehog signaling upon ROC1 knockdown, while blockage of SUFU expression restored the hedgehog signaling suppression triggered by the ROC1 knockdown, indicating that the ROC1-regulated hedgehog signaling in bladder cancer was dependent on SUFU degradation. One conceivable explanation may be that with the cooperation of ROC1, the F-box protein Fbxl17 binds to SUFU and promotes degradation in the CRL complex. Moreover, we also observed that silencing of both ROC1 and SUFU expression only partially restored the hedgehog pathway activity, indicating that the SUFU–Gli2 axis is necessary but not sufficient for regulation of hedgehog activity and that other regulatory pathways may also be involved.

In addition, our current ex vivo data further revealed an association between ROC1 expression and the hedgehog pathway proteins, i.e., ROC1 expression was inversely associated with SUFU (*P* < 0.001) but positively associated with Gli2 expression (*P* < 0.001) in bladder cancer tissues. We speculate that ROC1 could be a promising prognostic biomarker for predicting bladder cancer progression. The small molecule MLN4924 (Pevonedistat), which abrogates neddylation of the cullin subunit of CRLs, has been identified as a promising anticancer drug [6]. A recent study has shown that MLN4924 is able to synergistically enhance cisplatin cytotoxicity to urothelial carcinoma [26]. Our current study revealed that knockdown of ROC1 expression induced the same effect as MLN4924; therefore, we hypothesized that the combination of cisplatin with hedgehog inhibition by ROC1 silencing would provide a novel strategy to control bladder cancer in the future.

The development of bladder cancer, like most other human cancers, is a multifactorial and multistage cell transformation and carcinogenic process. Nevertheless, the current study does have some limitations. For example, besides the orthotopic tumour xenograft, we also observed lung and liver metastases in the in vivo experiment. This finding may be attributed to long of an observation period of three months, and distant metastasis could not be completely ruled out. Secondly, our sample size in the current study was small, and the association between ROC1 expression and patient prognosis was not investigated. Therefore, a future study with a larger sample size and follow-up data is needed to verify our current data. The present study is just a proof-of-principle study, and more research is needed to better understand the molecular mechanisms.

## Conclusion

Our current data demonstrate a novel mechanism by which ROC1 plays a pivotal role in bladder cancer progression by regulating the hedgehog-signaling pathway. Thus, ROC1 could be a novel anticancer target for bladder cancer therapy, although the precise underlying molecular mechanisms need to be further investigated.

## Supplementary Information

**Additional file 1: Figure S1.** ROC expression in ROC1 cDNA- or siRNA-transfected bladder cancer 5637 (A) and T24 (B) cells. Tumor cells were grown and stably transfected with ROC1 cDNA or transiently transfected with ROC1 siRNA and then subjected to western blot analysis of ROC1 protein.

**Additional file 2: Figure S2.** Knockdown of ROC1 led to cancer cell arrest at the G2/M phase. Bladder cancer 5637 (A) and T24 (B) cells were transfected with siROC1 and stained with propidium iodide for flow cytometric analysis. The representative images are shown in the left panel, and the quantified data are shown below. Representative results of three independent experiments are shown as means ± SEM; **P < 0.01, ***P < 0.001.

**Additional file 3: Figure S3.** Immunostaining of Gli2 protein in ROC1-knocked down or ROC1-overexpressed 5637 and T24 cells. Bladder cancer 5637 (A) and T24 (B) cells were grown and transfected with siROC1 or pROC1, stained with Gli2 (Red), and reviewed under a confocal microscope. The nuclear DNA was stained with 4,6-diamidino-2-phenylindole (blue). Scale bar, 10 µm.

**Additional file 4: Figure S4.** Knockdown of SUFU expression rescued tumor cell growth upon ROC1 knockdown. A cell viability assay was performed to assess the cell viability of 5637 cells transfected with siRNA targeting SUFU, ROC1, or both. Representative results of three independent experiments are shown as means ± SEM; *P < 0.05, **P < 0.01.

**Additional file 5: Figure S5.** Detection of Gli1 or Gli2 binding to SUFU in 5637 and T24 cells. Immunoprecipitation of SUFU from 5637 cells (A) and T24 cells (B). Nonspecific rabbit immunoglobulin G (IgG) was used as a negative control. Cell lysates were subjected to western blot analysis.

**Additional file 6: Figure S6.** MLN4924 regulation of bladder cancer cell growth. (A and B) Cell viability assay. Bladder cancer 5637 (A) and T24 (B) cells were grown and treated with different concentrations of the CRL inhibitor MLN4924 and then subjected to a cell viability assay. (C and D) Colony formation assay. Bladder cancer 5637 (C) and T24 (D) cells were grown and treated with different concentrations of the CRL inhibitor MLN4924 and then subjected to a colony formation assay. Representative results of three independent experiments are shown as means ± SEM; *P < 0.05, ***P < 0.001.

## Data Availability

The datasets used and/or analyzed during the current study are available from the corresponding author upon request.
