# Supplementary figures and images for: Regulator of cullins-1 (ROC1) negatively regulates the Gli2 regulator SUFU to activate the hedgehog pathway in bladder cancer
Source: Cancer Cell Int. 2021 Jan 26;21:75. doi: 10.1186/s12935-021-01775-5 (PMC7836478; doi:10.1186/s12935-021-01775-5)

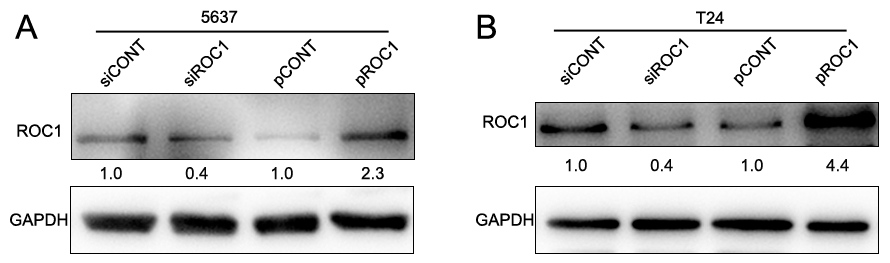

Supplement: Supplementary file 1 — Additional file 1: Figure S1. ROC expression in ROC1 cDNA- or siRNA-transfected bladder cancer 5637 (A) and T24 (B) cells. Tumor cells were grown and stably transfected with ROC1 cDNA or transiently transfected with ROC1 siRNA and then subjected to western blot analysis of ROC1 protein. [file 12935_2021_1775_MOESM1_ESM.tif]

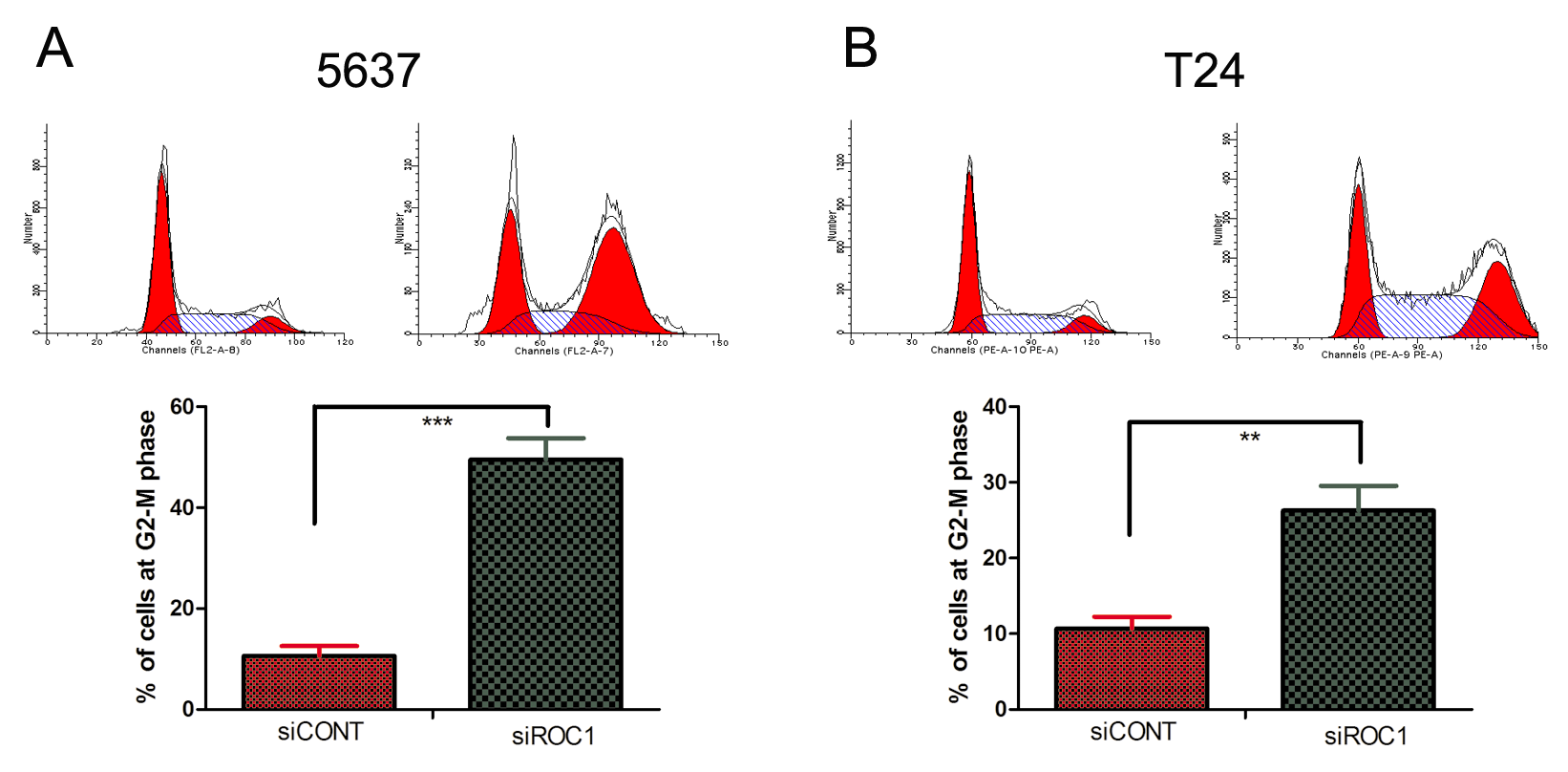

Supplement: Supplementary file 2 — Additional file 2: Figure S2. Knockdown of ROC1 led to cancer cell arrest at the G2/M phase. Bladder cancer 5637 (A) and T24 (B) cells were transfected with siROC1 and stained with propidium iodide for flow cytometric analysis. The representative images are shown in the left panel, and the quantified data are shown below. Representative results of three independent experiments are shown as means ± SEM; **P < 0.01, ***P < 0.001. [file 12935_2021_1775_MOESM2_ESM.tif]

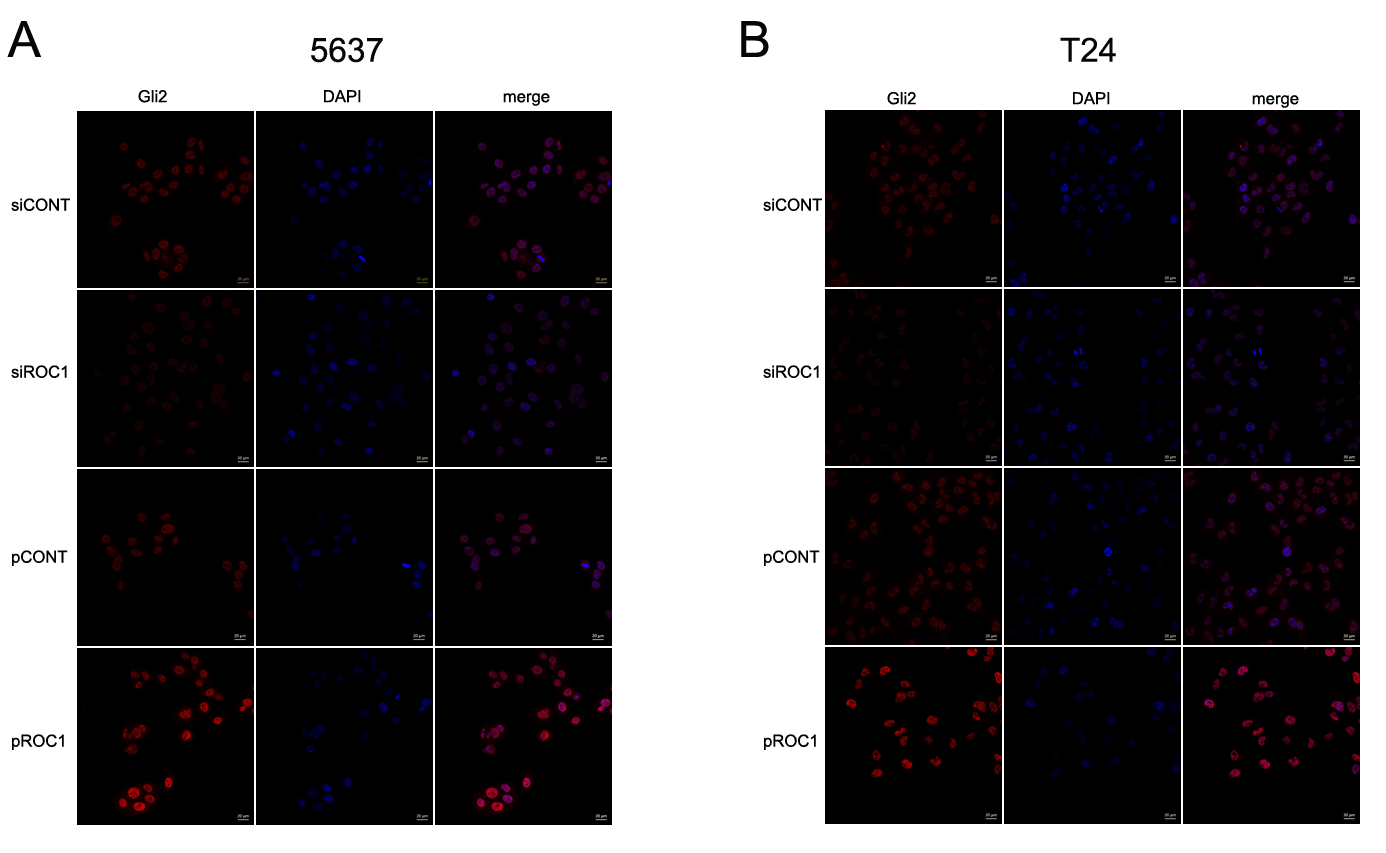

Supplement: Supplementary file 3 — Additional file 3: Figure S3. Immunostaining of Gli2 protein in ROC1-knocked down or ROC1-overexpressed 5637 and T24 cells. Bladder cancer 5637 (A) and T24 (B) cells were grown and transfected with siROC1 or pROC1, stained with Gli2 (Red), and reviewed under a confocal microscope. The nuclear DNA was stained with 4,6-diamidino-2-phenylindole (blue). Scale bar, 10 µm. [file 12935_2021_1775_MOESM3_ESM.tif]

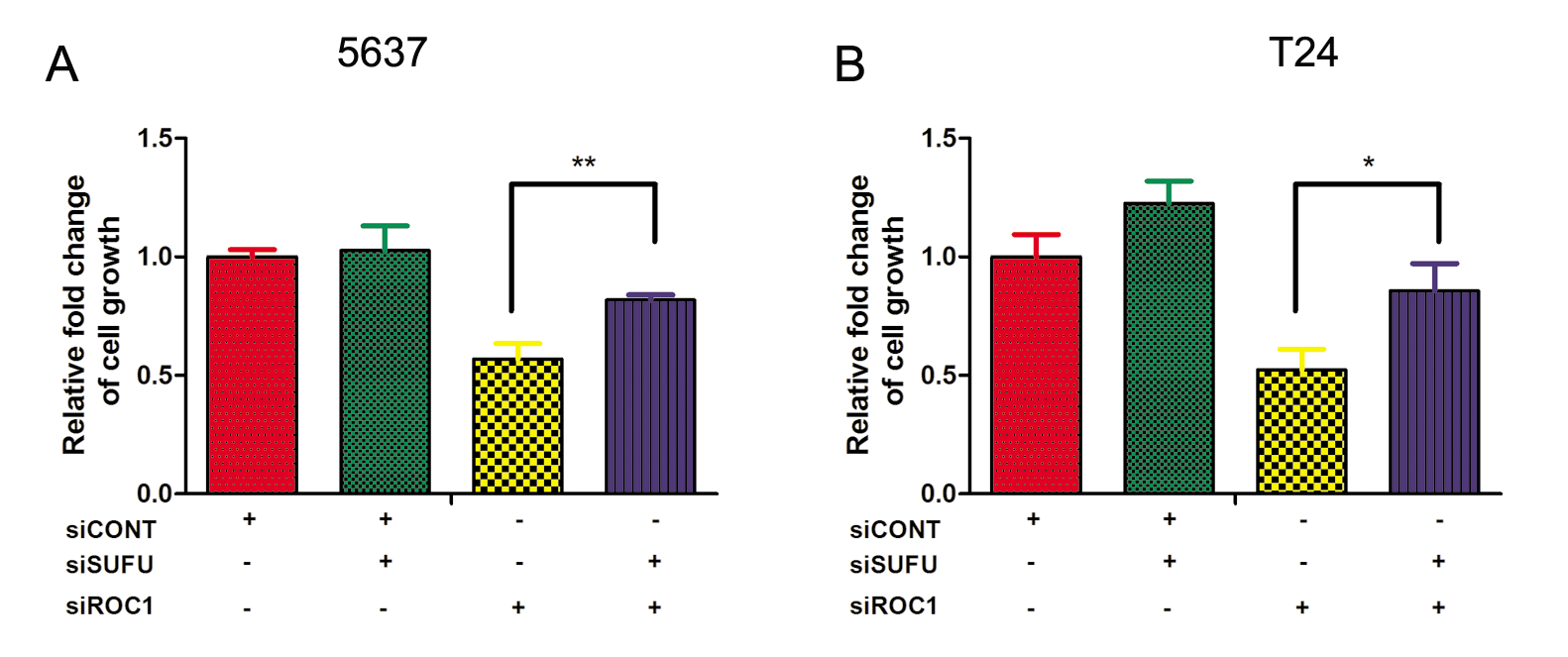

Supplement: Supplementary file 4 — Additional file 4: Figure S4. Knockdown of SUFU expression rescued tumor cell growth upon ROC1 knockdown. A cell viability assay was performed to assess the cell viability of 5637 cells transfected with siRNA targeting SUFU, ROC1, or both. Representative results of three independent experiments are shown as means ± SEM; *P < 0.05, **P < 0.01. [file 12935_2021_1775_MOESM4_ESM.tif]

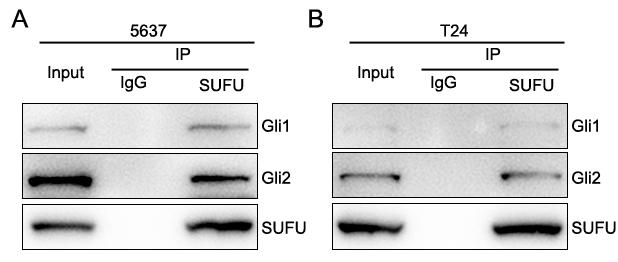

Supplement: Supplementary file 5 — Additional file 5: Figure S5. Detection of Gli1 or Gli2 binding to SUFU in 5637 and T24 cells. Immunoprecipitation of SUFU from 5637 cells (A) and T24 cells (B). Nonspecific rabbit immunoglobulin G (IgG) was used as a negative control. Cell lysates were subjected to western blot analysis. [file 12935_2021_1775_MOESM5_ESM.tif]

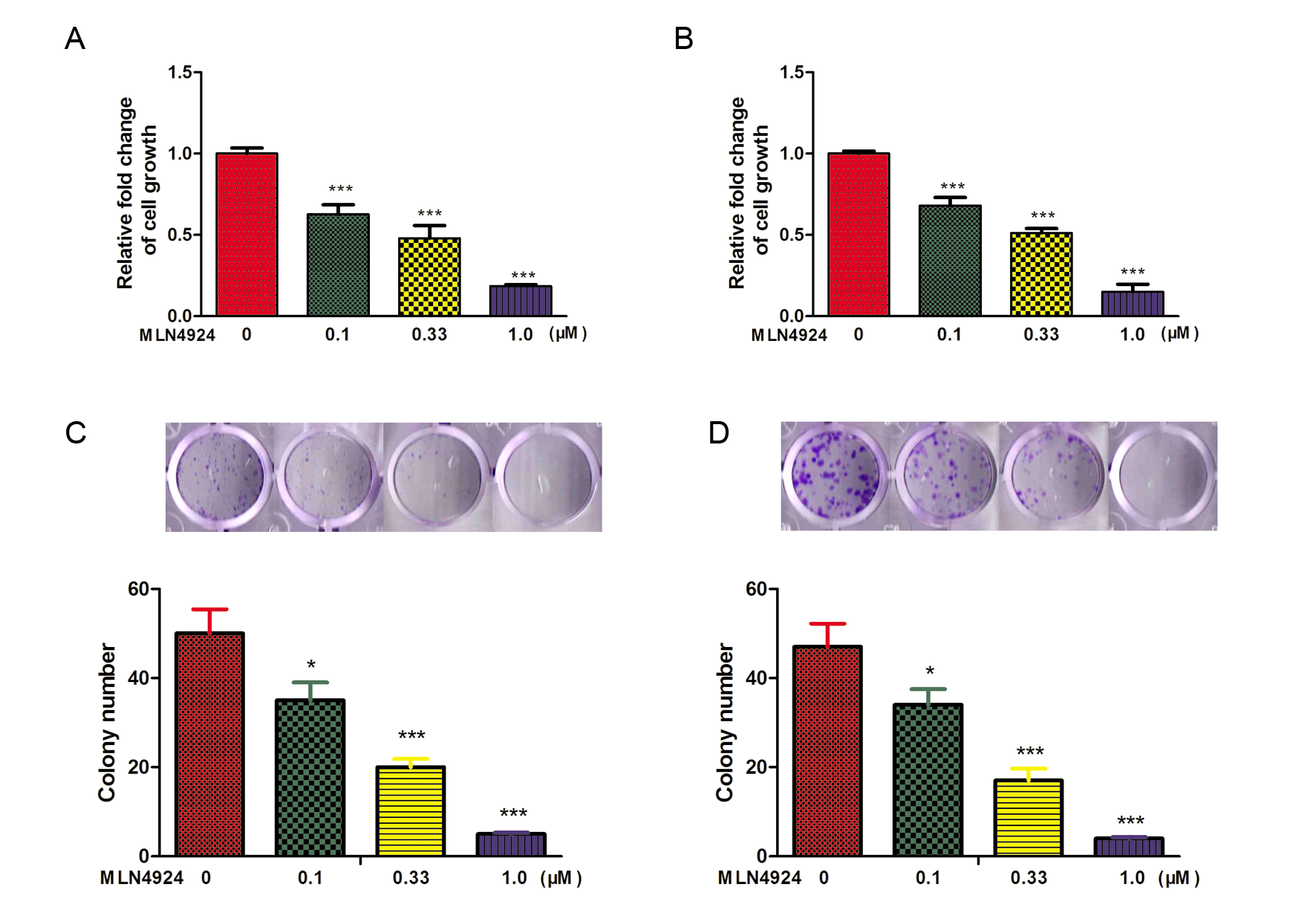

Supplement: Supplementary file 6 — Additional file 6: Figure S6. MLN4924 regulation of bladder cancer cell growth. (A and B) Cell viability assay. Bladder cancer 5637 (A) and T24 (B) cells were grown and treated with different concentrations of the CRL inhibitor MLN4924 and then subjected to a cell viability assay. (C and D) Colony formation assay. Bladder cancer 5637 (C) and T24 (D) cells were grown and treated with different concentrations of the CRL inhibitor MLN4924 and then subjected to a colony formation assay. Representative results of three independent experiments are shown as means ± SEM; *P < 0.05, ***P < 0.001. [file 12935_2021_1775_MOESM6_ESM.tif]
